# MicroRNA-195 Inhibits the Proliferation of Human Glioma Cells by Directly Targeting Cyclin D1 and Cyclin E1

**DOI:** 10.1371/journal.pone.0054932

**Published:** 2013-01-28

**Authors:** Wang Hui, Lu Yuntao, Luo Lun, Li WenSheng, Liang ChaoFeng, He HaiYong, Ba Yueyang

**Affiliations:** 1 Department of Neurosurgery, The Third Affiliated Hospital, Sun Yat-Sen University, Guangzhou, Guangdong, China; 2 Department of Neurosurgery, Nanfang Hospital, The First Affiliated Hospital of Southern Medical University, Guangzhou, Guangdong, China; H.Lee Moffitt Cancer Center & Research Institute, United States of America

## Abstract

Glioma proliferation is a multistep process during which a sequence of genetic and epigenetic alterations randomly occur to affect the genes controlling cell proliferation, cell death and genetic stability. microRNAs are emerging as important epigenetic modulators of multiple target genes, leading to abnormal cellular signaling involving cellular proliferation in cancers.In the present study, we found that expression of miR-195 was markedly downregulated in glioma cell lines and human primary glioma tissues, compared to normal human astrocytes and matched non-tumor associated tissues. Upregulation of miR-195 dramatically reduced the proliferation of glioma cells. Flow cytometry analysis showed that ectopic expression of miR-195 significantly decreased the percentage of S phase cells and increased the percentage of G1/G0 phase cells. Overexpression of miR-195 dramatically reduced the anchorage-independent growth ability of glioma cells. Furthermore, overexpression of miR-195 downregulated the levels of phosphorylated retinoblastoma (pRb) and proliferating cell nuclear antigen (PCNA) in glioma cells. Conversely, inhibition of miR-195 promoted cell proliferation, increased the percentage of S phase cells, reduced the percentage of G1/G0 phase cells, enhanced anchorage-independent growth ability, upregulated the phosphorylation of pRb and PCNA in glioma cells. Moreover, we show that miR-195 inhibited glioma cell proliferation by downregulating expression of cyclin D1 and cyclin E1, via directly targeting the 3′-untranslated regions (3′-UTR) of cyclin D1 and cyclin E1 mRNA. Taken together, our results suggest that miR-195 plays an important role to inhibit the proliferation of glioma cells, and present a novel mechanism for direct miRNA-mediated suppression of cyclin D1 and cyclin E1 in glioma.

## Introduction

The cyclins and their catalytic partners, the cyclin dependent kinases (CDKs), are cell cycle regulators. Cyclins act in concert with their CDKs to drive cells from one phase of the cell cycle to the next [1]. The first functions of cyclin D1 and cyclin E1 to be identified were related to control of G1-S phase cell cycle progression [2]. Cyclin D1 and cyclin E1 are thought to promote progression to the G1 phase of the cell cycle, on the basis of their cyclic pattern of mRNA expression, with maximal expression levels detected near the G1/S boundary [3]–[5]. During the G1 phase, the cyclin D1/CDK4 complex is phosphorylated by CDK-activating kinase (CAK). In turn, activated CDK4 is targeted by cyclin D1 and can hyperphosphorylate the tumor suppressor protein retinoblastoma (pRb) [6]–[7]. Phosphorylation of pRb leads to dissociation of the E2 promoter-binding protein dimerization partners (E2F) from the pRb/E2F complex, and dissociated E2F induces transcription of cyclin E1, which is required for entry to the S phase of the cell cycle [7].

The functions of cyclin D1 and cyclin E1 link the cell cycle to proliferation, apoptosis, invasion, differentiation and angiogenesis [8]–[12]. Therefore, cyclin D1 and cyclin E1 are considered to be key oncogenes. In agreement with their roles as oncogenes, cyclin D1 and cyclin E1 are overexpressed in breast, liver, lung and brain cancers [13]–[16]. However, the mechanisms by which cyclin D1 and cyclin E1 are upregulated in cancer cells remain to be fully elucidated.

MicroRNAs (miRNA) are small, non-coding 21–23 nucleotide RNAs which regulate gene expression by binding to the 3′-unstranslated regions of their target mRNA molecules, to repress transcription or induce mRNA degradation [17]–[18]. miRNAs have been demonstrated to play important roles in a wide variety of oncogenic activities, such as proliferation, angiogenesis, apoptosis, invasion and metastasis [19]–[22]. While the molecular mechanisms of miRNA-mediated gene regulation are still under investigation, recent studies have suggested that miRNA expression signatures are diagnostically and/or prognostically useful in human cancers.

Glioma, arising from glial cells, remains one of the most aggressive primary central nervous system (CNS) tumors. In spite of significant improvements in neurosurgery, radiotherapy and chemotherapy, the median survival time of high-grade glioma patients has remained at 12–15 months over the past decade, and the cumulative 1-year survival rate remains lower than 30% [23]–[28]. The poor prognosis of gliomas is largely attributed to their rapid growth, invasive/migratory nature and high rate of recurrence [29]–[31]. Although both genetic and environmental factors are considered to be major causes, the miRNA-based pathogenic mechanisms for glioma remains incompletely understood. Therefore, idenfication of microRNAs, whose deregulation would lead to development and progression of gliomas, is the the key to develop prognostic markers and effective therapeutic strategies.

In the present study, we report that miR-195 was significantly downregulated in glioma cells and clinical glioma tissues, compared to normal human astrocytes (NHA) and non-tumor associated tissues. We demonstrated that miR-195 promotes glioma cell proliferation by directly targeting the 3′-UTRs of cyclin D1 and cyclin E1, consequently reducing phosphorylation of pRb and downregulating the proliferative marker PCNA. Our results suggest that downregulation of miR-195 plays an important role in enhancing the proliferation of glioma cells.

## Materials and Methods

### Ethics Statement

For the use of clinical materials for research purposes, samples were obtained with prior written informed consents from the patients and approval from the Institutional Research Ethics Committees of Sun Yat-sen University and its Third Affiliated Hospital. Glioma cell lines A172, LN340, U118MG, LN464, SNB19, LN18, T98G, U251MG and LN235 were purchased from American Type Culture Collection (Manassas, VA).

### Cell Culture

Primary normal human astrocytes (NHA) were purchased from the Sciencell Research Laboratories (Carlsbad, CA) and cultured under the conditions as instructed by the manufacturer. Glioma cell lines A172, LN340, U118MG, LN464, SNB19, LN18, T98G, U251MG and LN235 were grown in DMEM medium supplemented with 10% fetal bovine serum (FBS). Cells were maintained in a humidified atmosphere at 37°C with 5% CO_2_.

### Plasmid, siRNA and Transfection

Cyclin D1 and Cyclin E1 ORF, without 3′UTR, were generated by PCR and cloned into pCDNA3.1 (+) vector (Invitrogen). The primers selected were as the following: Cyclin D1-ORF-up, 5′-GCCGAATTCACCATGGAACACCAGCTCCTGTG-3′; Cyclin D1-ORF-dn, 5′-GCCCTCGAGTCAGATGTCCACGTCCCGCA-3′; Cyclin E1-ORF-up, 5′-GCCGAAT TCACCATGCCGAGGGAGCGCAGGGA-3′; Cyclin E1-ORF-dn, 5′-GCCCTCGAGTCA CGCCATTTCCGGCCCGC-3′.

The region of human cyclin D1 3′UTR, from 1824 to 2236, and cyclin E1 3′UTR, from 126 to 508, generated by PCR amplification from DNA of the LN18 cells, were cloned into pEGFP-C1 (Clontech, Mountain View, CA) and pGL3 vector (Promega, Madison, WI). The primers selected were as the following: Cyclin D1-3′UTR-GFP-up, 5′-GCCCTCGAGCTT- GATGTTGAAGGGAGGTGGCA-3′; Cyclin D1-3′UTR-GFP-dn, 5′-GCCGGTACCATG- GCTAAGTGAAGCATGAGG-3′; Cyclin E1-3′UTR-GFP-up, 5′-GCCCTCGAGCTTGA- AAGTATTTCTGTGGATGG-3′; Cyclin E1-3′UTR-GFP-dn, 5′-GCCGGTACCAAAAAA- TGGATAGATATAGC-3′; Cyclin D1-3′UTR-luc-up, 5′-GCCCCGCGGTGTTGAAGGG- AGGTGGCA-3′; Cyclin D1-3′UTR-luc-dn, 5′-GCCCTGCAGATGGCTAAGTGAAGCAT- GAGG-3′; Cyclin E1-3′UTR-luc-up, 5′-GCCCCGCGGAAGTATTTCTGTGGATGG-3′; Cyclin E1-3′UTR-luc-dn, 5′-GCCCTGCAGAAAAAATGGATAGATATAGC-3′; siRNA used were: Cyclin D1 siRNA: CCACAGAUGUGAAGUUCAUUU; Cyclin E1 siRNA: CACCCUCUUCUGCAGCCAAUU. The miR-195 mimics, negative control and miR-195 inhibitor were purchased from RiboBio (Guangzhou, Guangdong, China). Transfection of the plasmids, siRNAs, microRNA and microRNA inhibitor was performed using Lipofectamine 2000 (Invitrogen) according to the manufacturer’s instructions.

### Western Blotting

Western blotting analysis was performed according to standard methods as previously described [32], using anti-PCNA, anti-cyclin D1, anti-cyclin E1, anti-CDK2, anti-phospho-CDK2, anti-p21, anti-GFP antibodies (Cell Signaling, Danvers, MA), and anti-pRb, anti-phosphor-pRb antibodies (Abcam, Cambridge, MA, USA). The membranes were stripped and re-probed with an anti-α-tubulin antibody (Sigma, Saint Louis, MI, USA) as a loading control.

### RNA Extraction and Real-Time Quantitative PCR

Total miRNA from cultured cells and fresh surgical glioma tissues was extracted using the mirVana miRNA Isolation Kit (Ambion, Austin, TX, USA) according to the manufacturer’s instructions. cDNA was synthesized from 5 ng of total RNA using the TaqMan miRNA reverse transcription kit (Applied Biosystems, Foster City, CA), and the expression levels of miR-195 were quantified using miRNA-specific TaqMan MiRNA Assay Kit (Applied Biosystems) on the Applied Biosystems 7500 Sequence Detection system. The expression of miRNA was defined based on the threshold cycle (Ct), and relative expression levels were calculated as 2^−[(Ct of miR−195) – (Ct of U6)]^ after normalization with reference to expression of U6 small nuclear RNA.

### 3-(4, 5-Dimethyl-2-thiazolyl)-2, 5-diphenyl-2H-tetrazolium Bromide (MTT) Assay

Cells, seeded on 96-well plates, were stained at indicated time points with 100 µl sterile MTT dye (0.5 mg/ml, Sigma) for 4 h at 37°C, followed by removal of the culture medium and addition of 150 µl of dimethyl sulphoxide (DMSO) (Sigma, St. Louis, MO, USA). The absorbance was measured at 570 nm, with 655 nm as the reference wavelength. All experiments were performed in triplicate.

### Anchorage-independent Growth Assay

Cells were trypsinized and 500 cells were resuspended in 2 ml complete medium plus 0.3% agar (Sigma, St Louis, MO). The agar–cell mixture was plated on top of a bottom layer consisting of 1% agar in complete medium. After 10 days, colony size was measured using an ocular micrometer and colonies larger than 0.1 mm in diameter were counted. The experiment was performed three times for each cell line.

### Bromodeoxyuridine Labeling and Immunofluorescence

Cells grown on coverslips (Fisher, Pittsburgh, PA) were incubated with bromodeoxyuridine (BrdUrd) for 1 h and then stained with an anti-BrdUrd antibody (Upstate, Temecula, CA) according to the manufacturer’s instructions. Gray level images were acquired using a laser scanning microscope (Axioskop 2 plus, Carl Zeiss Co. Ltd., Jena, Germany).

### Luciferase Assays

Cells (3.5×10^4^) were seeded in triplicate in 24-well plates and allowed to settle for 24 h. 100 ng of pGL3-Cyclin D1-3′UTR (wt/mut), pGL3-Cyclin E1-3′UTR (wt/mut), or control-luciferase plasmid plus 1 ng pRL-TK renilla plasmid (Promega, Madison, WI) were transfected into cells using Lipofectamine 2000 (Invitrogen Co., Carlsbad, CA) according to the manufacturer’s recommendations. Luciferase and renilla signals were measured 48 h after transfection using the Dual Luciferase Reporter Assay Kit (Promega, Madison, WI) according to the manufacturer’s protocol. Three independent experiments were performed and the data are presented as the mean ± SD.

### Flow Cytometry Analysis

Cells were harvested by trypsinization, washed in ice-cold PBS, and fixed in 80% ice-cold ethanol in PBS. Before staining, cells were sedimented in a chilled centrifuge and resuspended in cold PBS. Bovine pancreatic RNase (Sigma-Aldrich) was added to a final concentration of 2 µg/ml, and cells were incubated at 37°C for 30 min, followed by incubation with 20 µg/ml of propidium iodide (Sigma-Aldrich) for 20 min at room temperature. Cell cycle profiles of 5×10^4^ cells were analyzed using a FACSCalibur flow cytometer (BD Biosciences).

### Xenografted Tumor Model

BALB/c-nu mice (4–5 weeks of age, 18–20 g) were purchased from the Center of Experimental Animal of Guangzhou University of Chinese Medicine, and were housed in barrier facilities on a 12 h light/dark cycle. The BALB/c nude mice were inoculated subcutaneously with LN18-Vector (5×10^6^) in the left dorsal flank and with LN18-miR-195 (5×10^6^) in the right dorsal flank per mouse. Tumors were examined weekly; length, width, and thickness measurements were obtained with calipers and tumor volumes were calculated. Tumor volume was calculated using the equation (L*W^2^)/2. On day 35, then animals were euthanized, tumors were excised and weighed.

### Statistical Analysis

The two-tailed Student’s t-test was used to evaluate the significance of the differences between two groups of data in all pertinent experiments; A P values <0.05 was considered significant.

## Results

### miR-195 is Downregulated in Glioma Cell Lines and Glioma Tissues

**Figure 1:**
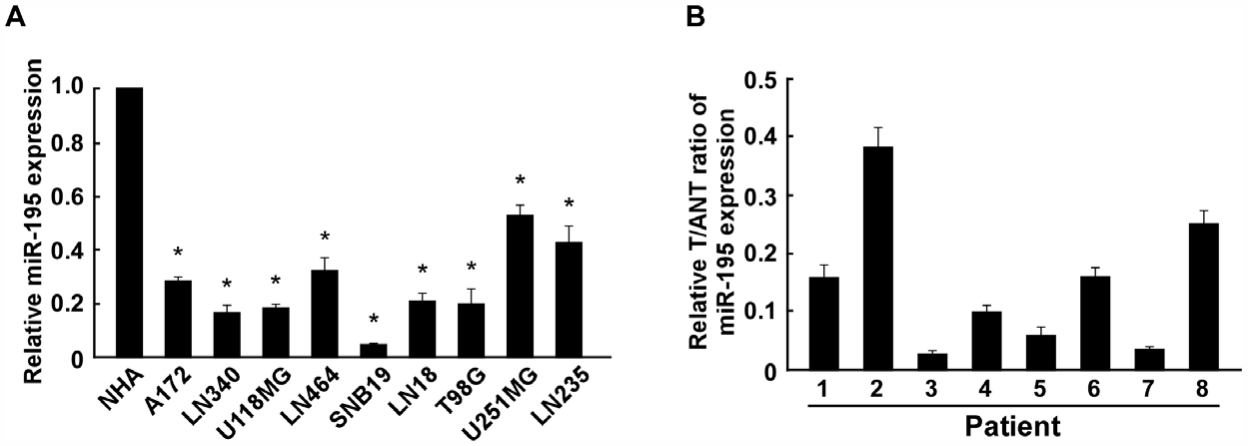
Analysis of miR-195 expression in glioma cell lines and tissues. A , Real-time PCR analysis of miR-195 expression in normal human astrocytes NHA and glioma cell lines, including A172, LN340, U118MG, LN464, SNB19, LN18, T98G, U251MG and LN235. The average miR-195 expression was normalized to U6 expression. **B**, The expression of miR-195 was examined in paired primary glioma tissues (T) and glioma adjacent nontumor tissues (ANT) from eight individual patients. The average miR-195 expression was normalized to U6 expression. Each bar represents the mean of three independent experiments. * *P*<0.05.

Real-time PCR analyses showed that expression of miR-195 was markedly lower in all eight analyzed glioma cell lines, including A172, LN340, U118MG, LN464, SNB19, LN18, T98G, U251MG and LN235, as compared with that in normal human astrocytes (NHA) (Figure 1A). To determine whether the miR-195 downregulation in glioma cell lines is also clinical relevant, we further examined the miR-195 expression in eight paired glioma tissues and adjacent nontumor tissues from the same patients. As shown in Figure 1B, comparative analysis showed that the expression level of miR-195 was also reduced in all 8 examined tumor tissues as compared to that in paired adjacent nontumor tissues. Collectively, our results indicate that miR-195 is downregulated in gliomas.

### miR-195 Overexpression Inhibits Proliferation of Glioma Cells

**Figure 2:**
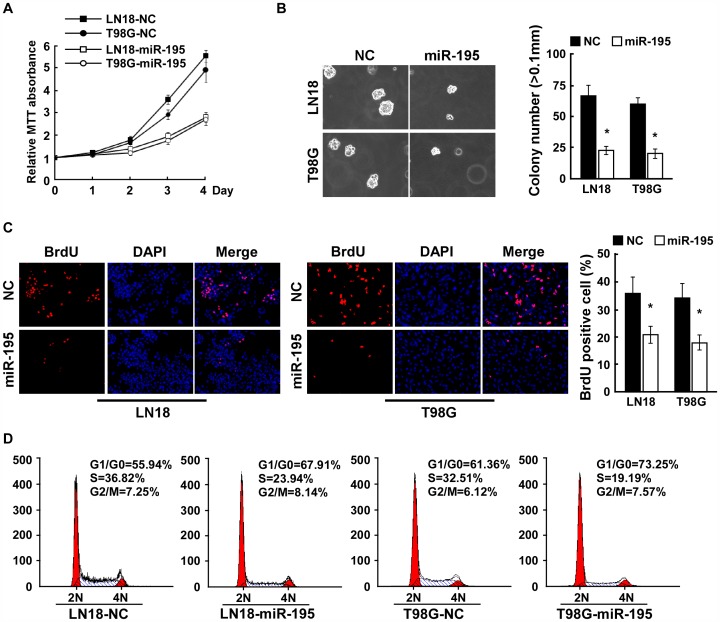
Upregulation of miR-195 suppresses the proliferation of glioma cells. A , MTT assays revealed that upregulation of miR-195 reduced cell growth of LN18 and T98G glioma cell lines, compared to negative control (NC)-transfected cells. **B**, Upregulation of miR-195 reduced glioma cell tumorigenicity as determined by anchorage-independent growth assay. Representative micrographs (left) and quantification of colonies that were larger than 0.1 mm (right) were scored. **C**, Representative micrographs (left) and quantification of BrdU incorporating-cells after transfection with negative control (NC) or miR-195. **D,** Flow cytometric analysis of indicated glioma cells after transfection with Negative control (NC) or miR-195. Each bar represents the mean of three independent experiments. * *P*<0.05.

To investigate the biological role of miR-195 in glioma progression, we first examined the effect of overexpressing miR-195 in glioma cells and relative miR-195 expression were analyzed using realtime PCR (Supplemental Figure 1). Strikingly, MTT assay showed that miR-195 overexpressing LN18 and T98G glioma cells exhibited significantly lower growth rates (approximately 2-fold lower) than control cells at day 4 after plating (Figure 2A). Furthermore, we found that ectopic expression of miR-195 dramatically reduced the anchorage-independent growth ability of LN18 and T98G glioma cells (Figure 2B), as indicated by reduced colony numbers and colony sizes. These results suggest that miR-195 upregulation inhibits proliferation of glioma cells.

To investigate the anti-proliferative effect of overexpressing miR-195 in glioma cells, BrdUrd incorporation assay was performed. As shown in Figure 2C, overexpression of miR-195 significantly decreased the percentage of BrdUrd-incorporating cells (21.3% vs 36.5% for LN18 cells, and 18.7% vs 34.2% for T98G cells), indicating that upregulation of miR-195 in LN18 and T98G glioma cells inhibits the DNA synthesis. Consistently, flow cytometry analysis showed that enforced expression of miR-195 not only significantly decreased the percentage of cells in the S peak but also increased the percentage of cells in the G1/G0 peak (Figure 2D), suggesting that miR-195 upregulation inhibits proliferation of glioma cells through induction of G1-S arrest.

### Inhibition of miR-195 Promotes Proliferation of Glioma Cells

**Figure 3:**
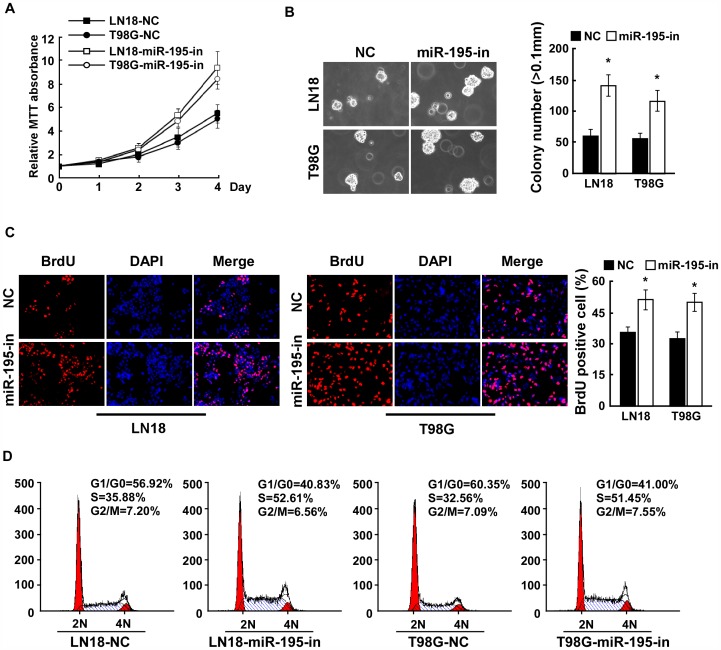
Inhibition of miR-195 promotes glioma cell proliferation. A , MTT assays revealed that inhibition of miR-195 promoted cell growth of glioma cell lines of LN18 and T98G. **B**, Inhibition of miR-195 promoted the anchorage-independent growth of glioma cells. Representative micrographs (left) and quantification of colonies that were larger than 0.1 mm (right) were scored. **C**, Representative micrographs (left) and quantification of BrdU incorporating-cells after transfection with NC or miR-195 inhibitor. **D,** Flow cytometric analysis of indicated glioma cells after transfection with NC or miR-195 inhibitor. Each bar represents the mean of three independent experiments. * *P*<0.05.

To further demonstrate the significance of the anti-proliferative function of miR-195 in glioma cells, the growth rate was examined in LN18 and T98G glioma cells transfected with miR-195 inhibitor. As shown in Figure 3A, inhibition of miR-195 dramatically increased the growth of LN18 and T98G glioma cells. Consistent with these results, the anchorage-independent growth ability of LN18 and T98G glioma cells transfected with the miR-195 inhibitor significantly increased, as indicated by increased colony number and size on soft agar (Figure 3B). Together, these results suggest that inhibition of miR-195 plays an important role in the proliferation and tumorigenic phenotype of glioma cells. Furthermore, we found that the percentage of S phase glioma cells dramatically increased in the miR-195 inhibited-glioma cells as compared with that in the control cells (Figure 3C). Moreover, flow cytometry assay displayed that the percentage of cells in the G1/G0 peak significantly decreased in response to miR-195 inhibition (Figure 3D). Collectively, our results suggest that inhibition of miR-195 promotes proliferation and the G1/S cell cycle transition in glioma cells.

### miR-195 Downregulates Cyclin D1 and Cyclin E1 by Directly Targeting their-3′UTRs

**Figure 4:**
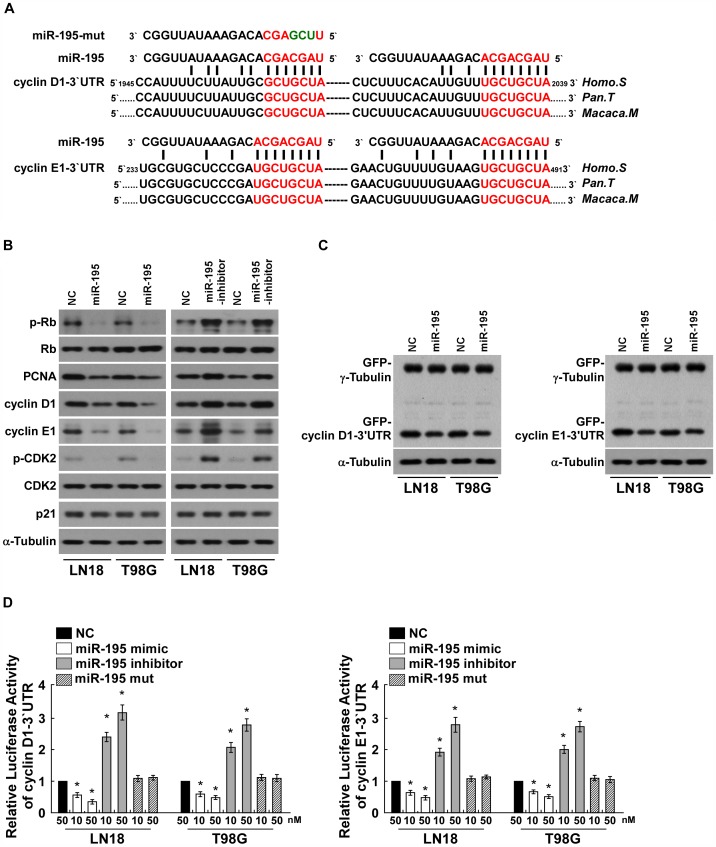
miR-195 downregulates cyclin D1 and cyclin E1 by directly targeting their 3 ′**-UTRs. A**, Predicted miR-195 target sequence in the 3′-UTR of cyclin D1 and cyclin E1 (cyclin D1 3′-UTR and cyclin E1 3′-UTR) and illustration of the three altered nucleotides in miR-195-mut. **B**, Western blotting analysis of expression of phosphorylated Rb (p-Rb), total Rb, PCNA, cyclin D1, cyclin E1, CDK2 and p21 in indicated cells. α-Tubulin served as the loading control. **C**, Western blotting analysis of GFP expression in indicated cells. **D**, Luciferase assay of indicated cells transfected with the pGL3-cyclin D1-3′UTR reporter (left) or the pGL3-cyclin E1-3′UTR reporter (right) with increasing amounts (10, 50 nM) of miR-195 mimic, or miR-195 inhibitor, or miR-195 mutant. Each bar represents the mean ± SD of three independent experiments. * *P*<0.05.

Western blotting analysis revealed that the phosphorylated pRb (p-pRb) and proliferative marker PCNA were also decreased in the miR-195-transfected cells and increased in the miR-195-inhibited cells, further demonstrating that miR-195 plays an important role in the proliferation of glioma cells (Figure 4B). Importantly, analysis using publicly available algorithms (TargetScan, Pictar, miRANDA) indicates that cyclin D1 and cyclin E1, key regulators of cell cycle, are the predicted targets of miR-195 (Figure 4A). As predicted, upregulation of miR-195 decreased, but inhibition of miR-195 increased, the expression levels of cyclin D1 and cyclin E1 in LN18 and T98G glioma cells (Figure 4B). However, dysregulation of miR-195 did not result in altered expression of another two cell cycle regulators, CDK2 and p21, which are not the predicted targets of miR-195, further demonstrating the specific effect of miR-195 on proliferation through targeting cyclin D1 and cyclin E1 (Figure 4B). Importantly, As shown in Figure 4C, ectopically expressing miR-195 only decreased the expression of GFP, which containing the 3′UTR of cyclin D1 or cyclin E1, but did not decreased the expression of GFP-γ-tubulin, indicating that miR-195 specifically affected the 3′UTR of cyclin D1 or cyclin E1 (Figure 4C). Furthermore, we found that overexpression of miR-195 reduced, but inhibition of miR-195 increased, the luciferase activity of cyclin D1-3′UTR or cyclin E1-3′UTR in a consistent and dose-dependent manner (Figure 4D). However, transfection of the miR-195-mut, containing mutations in the miR-195 seed region, did not decreased the luciferase activity of cyclin D1-3′UTR or cyclin E1-3′UTR (Figure 4D). Moreover, point mutations in the tentative miR-195-binding seed region in cyclinD1 3′-UTR and cyclinE1 3′-UTR abrogated the aforementioned repressive effect of miR-195 (Supplemental Figure 2A and 2B). Taken together, our results demonstrate that cyclin D1 and cyclin E1 are *bona fide* targets of miR-195.

To further investigate the role of Cyclin D1 and Cyclin E1 repression in miR-195-induced glioma cell growth arrest, we first examined the effects of Cyclin D1 and Cyclin E1 re-introduction on glioma cell proliferation. As predicted, re-introduction of the Cyclin D1 or Cyclin E1 ORF (without 3′UTR) in miR-195-transfected cells abrogated, at least partially, the miR-195-mediated glioma cell growth arrest, as analyzed by the BrdUrd incorporation assay. Importantly, co-expression of Cyclin D1 and Cyclin E1 indeed could further potentiate the proliferation of glioma cells, compared to that of Cyclin D1 or Cyclin E1 expressed alone (Supplemental Figure 2C). On the other hand, miR-195 inhibition-induced glioma cell growth promotion could be antagonized by individually silencing these two targets, and to further extent by co-silencing the two targets (Supplemental Figure 2D). Taken together, these results suggest that both Cyclin D1 and Cyclin E1 downregulations contribute to miR-195-induced glioma cell growth arrest.

### miR-195 Inhibits Glioma Proliferation in vivo

**Figure 5:**
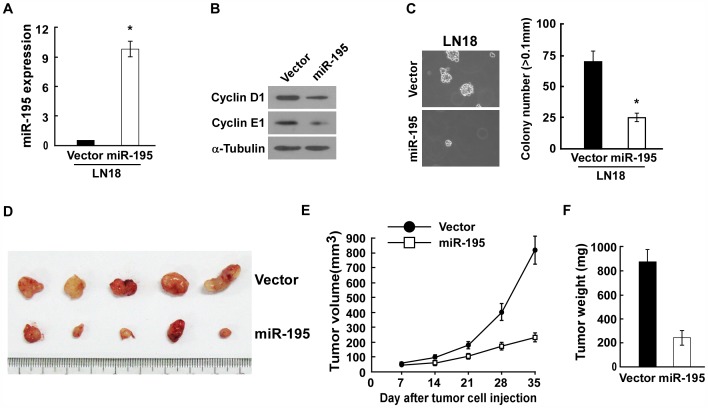
miR-195 inhibits glioma proliferation *in vivo*. A. Real-time PCR analysis of miR-195 expression in LN18-Vector and LN18-miR-195 stable cell lines. **B.** Western blotting analysis of Cyclin D1 and Cyclin E1 expression in vector- or miR-195-transduced cells. **C.** Representative micrographs (left panel) and quantification (right panel) of colonies determined by anchorage-independent growth assays. **D–F.** Xenograft model in nude mice. Indicated cells were injected into the dorsal flank of the mice. (**D**)**,** Images of the tumors from all mice. (**E**), Tumor volumes were measured on the indicated days. (**F**), Mean tumor weights.

To further investigate the effect of miR-195 reduction on glioma proliferation, the LN18 glioma cell lines, established to stably overexpress miR-195, were implanted into the right dorsal flank and with LN18-Vector cells in the right dorsal flank per mouse. As expected, the modified cells displayed upregulated miR-195, reduced Cyclin D1 and Cyclin E1 expression and presented lower growth rate *in vitro*. (Figure 5A–C). Importantly, consistent with the observed effects of the miR-195 *in vitro*, miR-195-overexpressing LN18 glioma cells formed much smaller tumors, about 0.27 fold of the control, indicating a dramatic inhibition of glioma cell proliferation by miR-195 *in vivo* (Figure 5D–F). Meanwhile, western blotting analysis confirmed that the expression levels of Cyclin D1 and Cyclin E1 in the tumors derived from LN18/miR-195 were significantly lower than those in the tumors derived from LN18/Vector (Supplemental Figure 3). Taken together, our results suggest that miR-195 inhibit glioma proliferation both *in vitro* and *in vivo* by repressing Cyclin D1 and Cyclin E1 expression.

**Figure 6:**
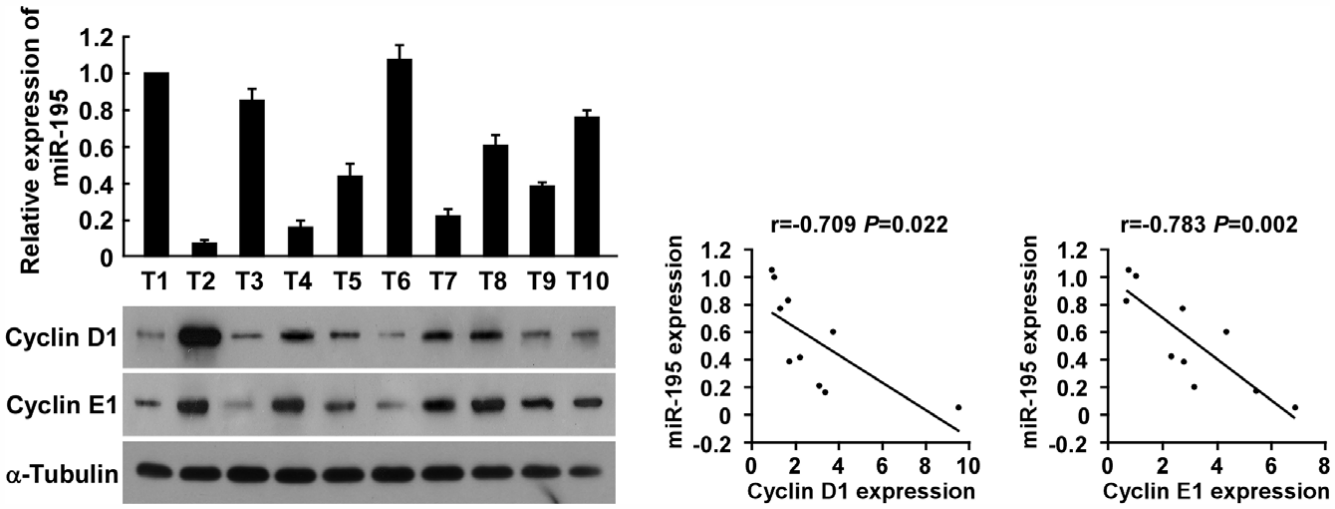
miR-195 expression inversely correlates with Cyclin D1 and Cyclin E1 expression in glioma tissues. Analysis (left) and correlation (right) between miR-195 expression and Cyclin D1 and Cyclin E1 expression levels in 10 freshly collected human glioma samples. Each bar represents the mean ± SD of three independent experiments.

Finally, we examined whether the low miR-195-induced Cyclin D1/Cyclin E1 upregulation identified by our study is clinically relevant. As shown in Figure 6, miR-195 levels in 10 freshly collected glioma samples inversely correlated with the expression of Cyclin D1 (r = −0.709, *P* = 0.022), and Cyclin E1 (r = −0.783, *P* = 0.002). Moreover, as shown in Supplemental Figure 4, the levels of Cyclin D1 and Cyclin E1 proteins were upregulated in glioma cell lines relative to normal astrocytes, and in tumors relative to adjacent normal tissue, which further confirm the notion that downregulation of miR-195 could lead to upregulation of Cyclin D1 and Cyclin E1 expression in glioma. Taken together, these results suggest that miR-195 can inhibit proliferation in gliomas, and that downregulation of miR-195 plays an important role in the progression of gliomas.

## Discussion

The key finding of the current study is that miR-195 expression is markedly downregulated in glioma cells and clinical glioma tissues as compared to NHA and normal brain tissues. Ectopic expression of miR-195 could decreased the proliferation and anchorage-independent growth of glioma cells, while inhibition of miR-195 reversed these effects. Moreover, we demonstrated that upregulation of miR-195 in glioma cells led to the downregulation of phosphorylated pRb and proliferative marker PCNA through downregulation of cyclin D1 and cyclin E1 via directly targeting the 3′-UTR of cyclin D1 and cyclin E1. Taken together, our results suggest that downregulation of miR-195 plays an important role in promoting carcinogenesis and progression of gliomas.

As members of the cell cycle regulator family, cyclin D1 and cyclin E1 have been recognized as oncogenes. It has been reported that the expression levels of cyclin D1 and cyclin E1 are related to the malignant degree of cancers, including glioma, breast cancer, bladder cancer, etc. [33]–[38]. Cyclin D1, cyclin E1 and pRb are known to orchestrate cell cycle progression, and functional interaction between cyclin D1, cyclin E1 and pRb plays an important role in cancer cell growth and tumorigenesis [39]–[40]. Cyclins form complexes with and function as regulatory subunits of the cyclin-dependent kinases (CDK), and cyclin/CDK complex activity is required for the G1/S cell cycle transition [39]–[40]. Cyclin/CDKs complexes phosphorylate and inactivate the tumor suppressor protein pRb, resulting in the transcriptional initiation of E2F-regulated genes, which are important for entering the S phase and for DNA replication [41]–[43]. Consequently, upregulation of cyclin D1 and cyclin E1 have been demonstrated to induce a rapid cell cycle transition and increase cell proliferation rate.

MicroRNAs, a class small regulatory RNA molecules that represse gene expression of their mRNA targets in a sequence-specific manner, have been identified as key regulators in a wide variety of oncogenic processes, such as cell proliferation, angiogenesis, cellular differentiation, invasion and metastasis, and can function as either tumor suppressors or oncogenes [26]–[31]. This study demonstrates that miR-195 was significantly downregulated in human glioma cell lines and glioma tissues, compared to the adjacent non-cancerous tissues. Furthermore, overexpression of miR-195 reduced glioma proliferation, tumorigenicity and cell cycle progression, whereas suppression of miR-195 enhanced proliferation, tumorigenicity and cell cycle progression. These results indicate that miR-195 may function as a tumor suppressor miRNA and downregulation of miR-195 may correlate with clinical progression in glioma. Consistent with these findings, miR-195 has been reported to be downregulated in a variety of different tumor types including hepatocellular carcinoma, gastric cancer and breast cancer [44]–[46]. Analysis of the GSE13030 dataset also revealed low expression of miR-195 in glioma tissues. Coincidently, Zhang QQ, et al. recently demonstrated that microRNA-195 could play a tumor-suppressor role in human glioblastoma cells by targeting E2F3 and CCND3 [47]. Combining Zhang’s work and ours, both two groups demonstrated that miR-195 downregulation promotes glioma cell proliferation. Strikingly, we both highlighted the point that one microRNA has multiple targets to perform its biological function, as found that miR-195 exhibited proliferation-inhibiting role by targeting Cyclin D1, Cyclin E1, E2F3 and CCND3.

Recently, accumulating evidence has indicated that expression of the cyclin family can be regulated by miRNAs. It was reported that miR-17, miR-20a and miR-106b coordinately regulate the expression of cyclin D1 by directly targeting its 3′-UTR, to affect cell cycle progression in unrestricted somatic stem cells [48]. Zhang W, et al. found that miR-520b is able to inhibit the growth of hepatoma cells by targeting MEKK2 or cyclin D1 both in vitro and in vivo [49]. Additionally, Ding and colleagues reported that upregulation of miR-29c downregulated cyclin E and suppressed its oncogenic activity in esophageal squamous cell carcinoma, without affecting other G1 phase-related proteins level, such as cyclin D1, cyclin D2 [50].

In this study, cyclin D1 and cyclin E1 were identified as theoretical targets of miR-195 using bioinformatic analysis. We were able to demonstrate that cyclin D1 and cyclin E1 are *bona fide* targets of miR-195 using different methods. Western blotting analysis confirmed that overexpression of miR-195 downregulated cyclin D1 and cyclin E1, and luciferase reporter assays demonstrated that miR-195 targeted cyclin D1 and cyclin E1 via the miR-195 binding sites in their 3′-UTRs. Furthermore, ectopic overexpression of miR-195 did not affect other cell cycle regulators (CDK2 and p21) which were not predicted to be targets of miR-195, further demonstrating that miR-195 affects proliferation by specifically targeting cyclin D1 and cyclin E1. Similar to our results in glioma, it was previously reported that miR-195 inhibited the hepatocellular carcinoma cells and hepatic stellate cells proliferation, respectively through targeting CyclinD1and CyclinE1 [51]–[52], indicating that the miR-195-downregulation-induced Cyclin D1/Cyclin E1 expression might be Universal among different types of tumors.

In summary, the current study provides the first evidence of an important link between miR-195 and proliferation in human glioma cells. Our findings suggest that miR-195 directly targets the 3′-UTR of the oncogenes cyclin D1 and cyclin E1 to inhibit proliferation by inducing G1-S arrest. Understanding the precise role played by miR-195 in the progression of glioma will increase our knowledge of tumor biology and explore the potential of miR-195 as a diagnostic/prognostic marker and novel therapeutic target for glioma.

## Supporting Information

Figure S1 **Real-time PCR analysis of miR-195 expression in LN18-NC, LN18-miR-195, T98G-NC, T98G-miR-195 transfected cells.** The average miR-195 expression was normalized to U6 expression. Each bar represents the mean of three independent experiments. * *P*<0.05.
(TIF)

Figure S2 **A. illustration of point mutations in the tentative miR-195-binding seed region in Cyclin D1 3′-UTR and Cyclin E1 3′-UTR.** **B**. Luciferase assay of indicated cells transfected with pGL3-Cyclin D1 (or E1)-3′UTR (mut) with miR-195 mimic oligonucleotides. **C–D**, Upper panel: BrdUrd incorporation in the indicated cells with different transfection. Lower panel: Western blotting analysis of Cyclin D1 and Cyclin E1 expression in indicated cells. α-tubulin was used as a loading control. Bars represents the mean ± SD of three independent experiments. * P, <0.05.
(TIF)

Figure S3 **Western blotting analysis of the expression levels of Cyclin D1 and Cyclin E1 in the tumors derived from LN18/Vector cells, or from LN18/miR-195 cells.** α-Tubulin was used as a loading control.
(TIF)

Figure S4 **A–B, Western blotting analysis of expression of cyclin D1, cyclin E1, in glioma cell lines (A) and paired patient tissue samples (B).** α-Tubulin served as the loading control.
(TIF)
