# Supplementary figures and images for: MicroRNA-195 Inhibits the Proliferation of Human Glioma Cells by Directly Targeting Cyclin D1 and Cyclin E1
Source: PLoS One. 2013 Jan 28;8(1):e54932. doi: 10.1371/journal.pone.0054932 (PMC3557299; doi:10.1371/journal.pone.0054932)

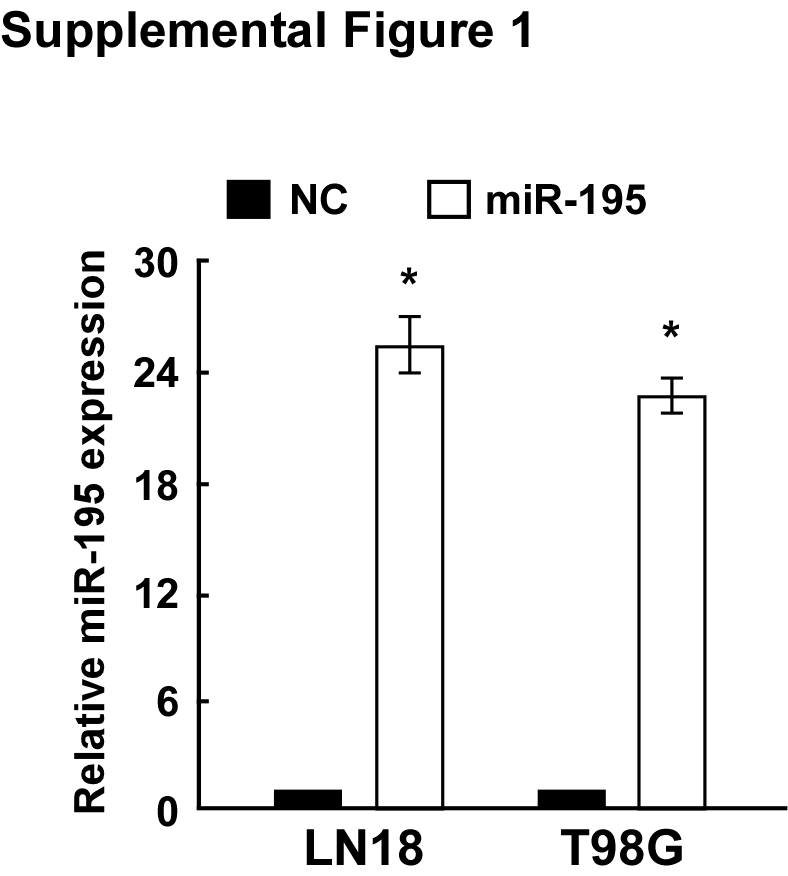

Supplement: Figure S1 — Real-time PCR analysis of miR-195 expression in LN18-NC, LN18-miR-195, T98G-NC, T98G-miR-195 transfected cells. The average miR-195 expression was normalized to U6 expression. Each bar represents the mean of three independent experiments. * P<0.05. (TIF) [file pone.0054932.s001.tif]

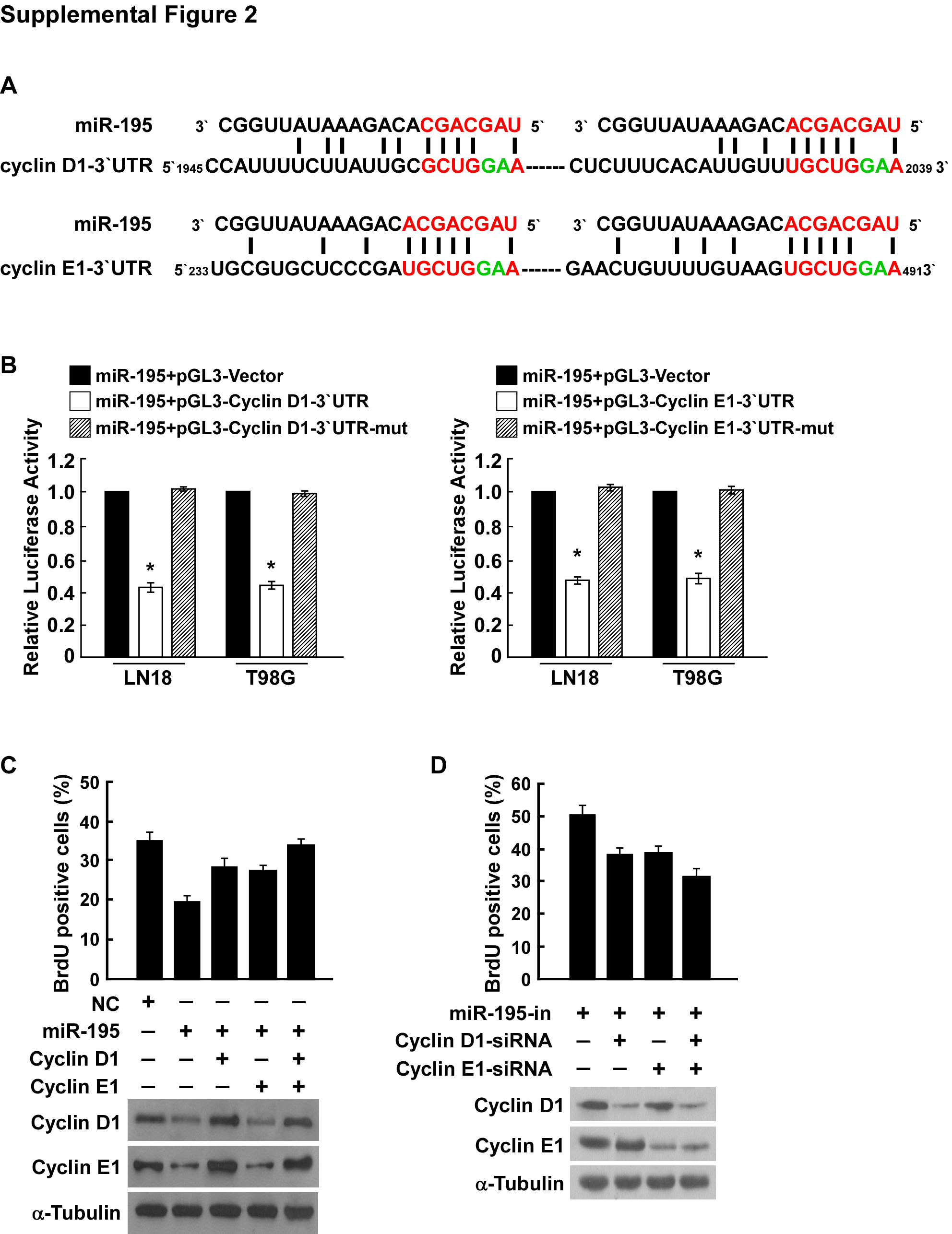

Supplement: Figure S2 — A. illustration of point mutations in the tentative miR-195-binding seed region in Cyclin D1 3′-UTR and Cyclin E1 3′-UTR. B. Luciferase assay of indicated cells transfected with pGL3-Cyclin D1 (or E1)-3′UTR (mut) with miR-195 mimic oligonucleotides. C–D, Upper panel: BrdUrd incorporation in the indicated cells with different transfection. Lower panel: Western blotting analysis of Cyclin D1 and Cyclin E1 expression in indicated cells. α-tubulin was used as a loading control. Bars represents the mean ± SD of three independent experiments. * P, <0.05. (TIF) [file pone.0054932.s002.tif]

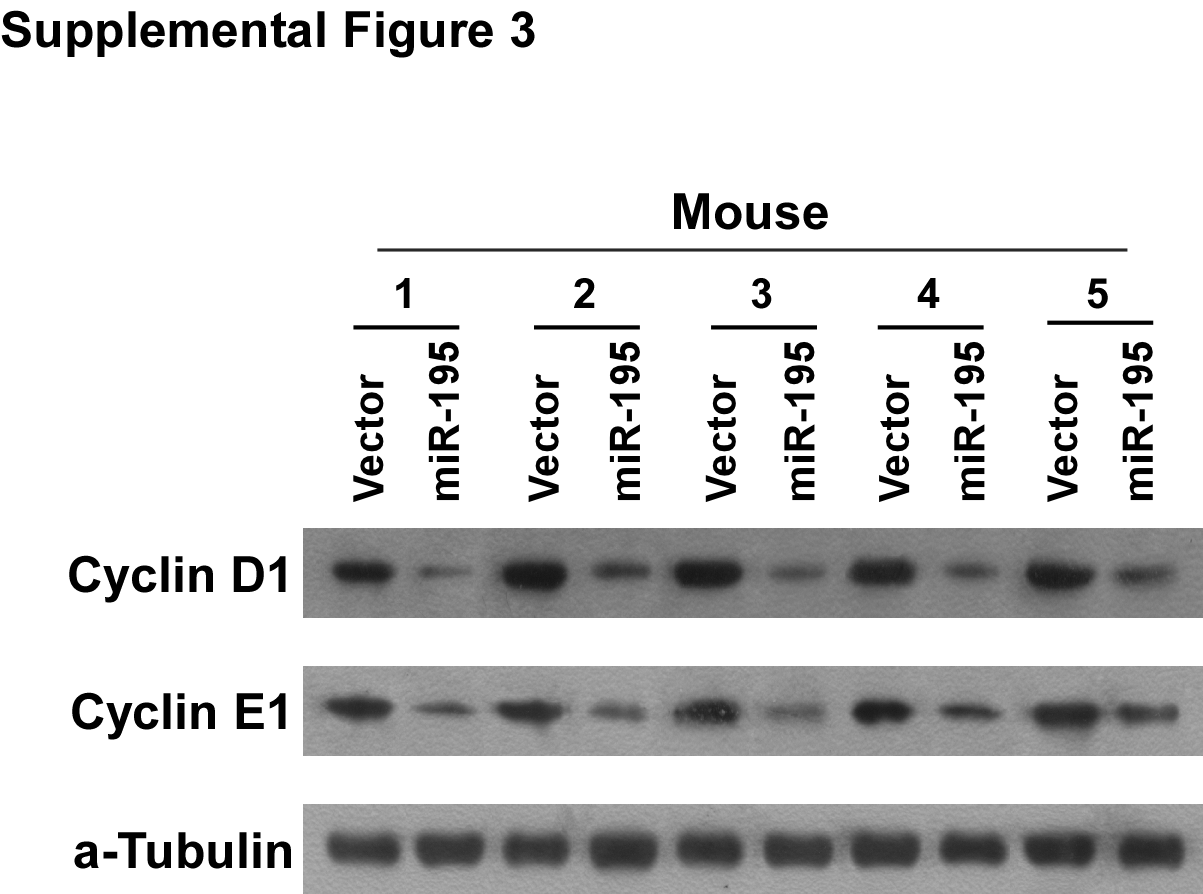

Supplement: Figure S3 — Western blotting analysis of the expression levels of Cyclin D1 and Cyclin E1 in the tumors derived from LN18/Vector cells, or from LN18/miR-195 cells. α-Tubulin was used as a loading control. (TIF) [file pone.0054932.s003.tif]

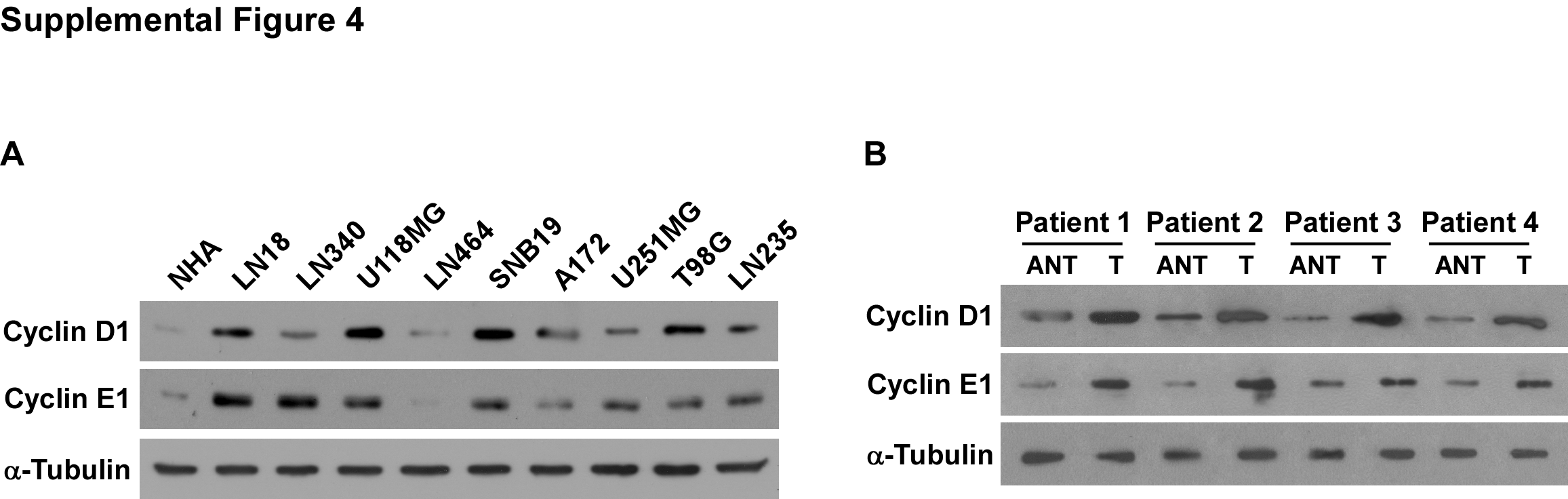

Supplement: Figure S4 — A–B, Western blotting analysis of expression of cyclin D1, cyclin E1, in glioma cell lines (A) and paired patient tissue samples (B). α-Tubulin served as the loading control. (TIF) [file pone.0054932.s004.tif]
